# Exploring health and toxicity in food choices: 10 examples navigating the gray area

**DOI:** 10.3389/fnut.2023.1301757

**Published:** 2024-01-05

**Authors:** Aalt Bast, Khrystyna O. Semen

**Affiliations:** ^1^University College Venlo, Maastricht University, Venlo, Netherlands; ^2^Department of Pharmacology and Toxicology, Faculty of Health, Medicine and Life Sciences, Maastricht University, Maastricht, Netherlands

**Keywords:** hormesis, toxicity, health, nutrition, flavonoid, taste, botanicals, vitamin B6

## Abstract

People’s perception on what is healthy and what is toxic food, determines food preferences and eating behavior. The difference between heathy and toxic food and food ingredients is however not always clear. This is illustrated with 10 examples. Unjustly, all-natural food is regarded as safe. Regulation on health claims on food and food risks is not balanced. Biphasic responses of the physiological effect of food ingredients show that mild toxicity of these substances results in health promotion. Nutritional substances with drugs may have either a negative or a positive effect on health. New toxicological methodologies can be brought into play, to better understand the dynamics of health and disease. Unfortunately, we still cannot taste toxicity.

## 1 Introduction

Tasters were important in ancient history. With some imagination, one might say that tasters can be regarded as early toxicologists. Tasters had to protect Egyptian pharaohs, and Roman emperors. To prevent poisoning of these rulers with food by competitors, tasters were hired. The bible tells a famous story of the Pharaoh who was angry with two of his servants, and put them in prison. One of them was the Pharaoh’s cupbearer. The other was the Pharaoh’s chief baker. On Pharaoh’s birthday,

“He [the Pharaoh] restored the chief cupbearer to his former position so that he placed the cup in Pharaoh’s hand, but the chief baker he impaled” [Genesis 40:1–23].

The chief baker and chief cupbearer were probably high priests and as servants of the Pharaoh of high social standing. The latter makes it attractive to compare them with modern toxicologists [smile authors]. The comparison can be extended. Food was regarded as the bearer of divinity and of life and was therefore given in the hand of the Pharaoh by these high priests. Our current view on food also changes from merely a necessity to survive to something that is the bearer of health. We now use fortified foods, food supplements and even nutraceuticals to boost our health. However, when this area is considered more closely, the distinction between healthy and toxic is far from clear. This is illustrated with 10 random examples.

## 2 Regulatory inconsistency for health promoting and toxic aspects of food

Regulators frequently use the precautionary principle to handle putative food risks. A well-known definition of precaution is the triple-negative definition, which reads “not having scientific certainty is not a justification for not regulating.” The principle reflects the impossibility to provide absolute proof of safety. The precautionary principle seems to offer guidance on what to regulate (1). However, by definition it does not. In order to implement this regulatory framework on toxic risks, random choices on risks to be tackled are made. Moreover, it blinds regulators for external effects of these choices. This way of regulation emphasizes risk. There is always a toxic risk of food.

Health promoting effects of food are regulated differently from toxic risks. A legal framework used to highlight a particular beneficial effect of a food product should ensure that a health claim is clear, accurate and based on scientific evidence. Information that is misleading to consumers is prohibited. The roadmap to a health claim automatically entails that well-designed placebo controlled double blind studies are necessary to authorize a health claim for a food product.

This shows a regulatory inconsistency in dealing with toxic aspects of food vs. a health claim on food. To balance this better, it should be considered to introduce the concept health risk, a graded health promoting response. This would be in alignment with the way in which toxic risks are presented (2).

## 3 Natural safety vs. chemical danger: biophilia and chemophobia

There is a wide spread belief that compounds derived from nature are not really chemicals. Or at least they do not count as a chemical, because the word “chemical” has a negative connotation. Substances that are made in plants, microbes or animals are just there by nature. We are part of nature and natural equals beneficial. Natural is frequently marketed with the prefix “bio.” Marketing uses this prefix eagerly. Biovitamin C sells better than just vitamin C (3). Conversely, synthetic substances are inherently regarded unhealthy. It is implied that the body is not equipped to cope with these man-made compounds. The fear or aversion to chemicals is called chemophobia and the intuitive love of all-natural, biophilia. The public sense of healthy food vs. toxic food is strongly influenced by these emotional responses.

A seminal paper documenting that natural is not synonymous with safety has been written by Ames et al. already in 1990 (4). The paper indicated that 99.99% (by weight) of the pesticides in the American diet are chemicals that plants produce to defend themselves. Comparative hazards of human exposures to synthetic pesticide residues are insignificant.

Statements that organic food is healthier are false. In fact there are many other examples illustrating that biophobia would be more appropriate than biophilia (5).

Unceasing information on chemicals (and it is without saying both of natural and of man-made origin) will aid general understanding of the role and use of these ingredients in food. Demystifying ingredients in food products will ultimately help to rationalize healthy and safety aspects of food.

## 4 New concepts of health: toxic changes lead to health

The WHO definition of health dates from 1948 and reads “A state of complete physical, mental and social well-being and not merely the absence of disease or infirmity.” It is not surprising that this all-encompassing definition emerged directly after the horrors of World War II. At that moment, everything should get better, including all aspects of health. Following this definition, powerful drugs were designed in the 20th century. Drugs with a specific action on a single molecular target, thus minimalizing side effects. Pharmacology, the science of drug action, was precisely described as a form of “selective toxicity” (6). Apparently, even in that period, a selective form of toxicity was regarded as a stimulating factor for health.

Increasing knowledge on molecular processes that define health suggests that health is not a stagnant condition, and the original definition of health has become flawed. Health forms a dynamic condition rather than a fixed state of perfection and can be seen as “the ability to adapt.” Obviously, this has consequences for preventing or treating diseases. Health promoting approaches should aim for increasing the aptitude to adapt.

Moreover, it is increasingly recognized that a shortcoming of a healthy homeostasis can involve various physiological pathways simultaneously, not just one specific molecular system. An imbalanced homeostasis may be the result of an intrinsic or extrinsic stressor. The increased ability to withstand such a stressor can then be regarded as a marker for health. To measure the health promoting effects of food, new methods should be developed. It is suggested that a mild aberrant physiology induced via an intrinsic stressor (e.g., a slight metabolic disturbance) or via an extrinsic stressor (e.g., a high caloric meal) might be used to investigate whether the intervention is able to increase the ability to adapt.

The other notion is that food in contrast to selectively acting drugs, works via a multitude of targets. In addition, the effects of food are generally less strong than those of drugs. This so-called pleiotropic action of food requires integrative methodologies for determining activity (7). A combination of markers could be envisioned to characterize a food effect. Several attempts have undertaken in this respect. Unfortunately, these new concepts are not yet incorporated in regulatory documents.

In conclusion, moderate toxic changes might shift the physiological balance in such a way that a beneficial health promoting increase in adaptation ability results.

## 5 Lessons from toxicology for health effects of food

In an inspiring paper, Langley et al. argued that current and future biomedical knowledge in the 21^st^ century will ultimately lead to understanding of the dynamics of human disease (8). Human specific models understanding disease pathways comparable to unraveling adverse outcome pathways (AOPs) in toxicology will aid to comprehend cause and progression of pathophysiology. AOPs describe how interactions of compounds with biological systems cause injury and thus AOPs are thought to construct non-animal testing strategies as predictive models for toxicity of compounds. Similarly, unraveling pathways of positive effects of food constituents will lead to understanding how these will lead to health promoting effect of food ingredients.

## 6 Hormesis: a biphasic response to toxic compounds

Following examples in paragraphs 4 and 5, automatically the process of hormesis comes to mind. Hormesis is best described as an adaptive response to low levels of stress or damage by, for example compounds, resulting in enhanced robustness of some physiological systems for a finite period (9). The dose–response relationship is one of the most important aspects in toxicology. Safety regulation is built around the theory of linear dose response relationships. Risk predictions based on animal experiments using high doses still are mainstay in toxicology. Extrapolation to lower dose, the dose of exposure, is subsequently performed. The assumption of a linear relationship between dose and response ignores that our cells have developed mechanisms to detoxify harmful chemicals. In fact, low doses of these chemicals may even trigger beneficial responses. These adaptive or biphasic responses are also known as hormetic responses. Although there are numerous examples of compounds that follow this hormetic response, toxicological thinking is still hesitant to apply it and to use the beneficial hormetic stimulus of “toxic” compounds to our advantage.

This hesitation is understandable since there are still many questions to be solved (9). Such as (i) what is the optimal dose, frequency, duration and timing of exposure; (ii) what are synergistic stimuli; (iii) what is the kinetics of the hormetic response; (iv) how is the response influenced by age or gender etc.; (v) are there any adverse effects. For now, this will hamper direct application of the hormetic response to promote health.

## 7 The example of flavonoids

In the beginning of the 20th century, several vitamins were discovered. From citrus fruits not only vitamin C but also vitamin P was isolated. The chemical characterization of vitamin P appeared to be difficult. Moreover, no deficiency disease could be linked to this component from citrus extract. It appeared however that this yellow colored pigment “vitamin P” had protective effects on vascular permeability and could enforce the effect of vitamin C. The citrus extract components were then named flavonoids [*flavus* (Latin) means yellow]. More specifically the major components of the citrus extract could be identified as oligomers of flavan-3-ol units, i.e., (+)-catechin or (−)-epicatechin (10). The average total flavan-3-ol intake in Europe has been reported to be 369 mg/d. There are many other flavonoids. Quercetin, commonly found in apples, onions and green tea forms 70% of the total flavonoid intake (11).

When the timeline of major achievements in the field of molecular biology, medicine and nutritional science is plotted, it is remarkable that flavonoids follow that line perfectly. This indicates the multitude of effect these flavonoids have. It started with the discovery of vitamins, and currently flavonoids are investigated as modulators of epigenetic processes.

A general comment on the use of these flavonoids is their apparent low bioavailability. However, there are indications that flavan-3-ols, known for their effect on microcirculatory vessels, have strong affinity for the vascular wall. Moreover, quercetin seems to cumulate to some extent in lung tissue (12). It is not surprising therefore that a beneficial effect of quercetin has been established in the lung disease sarcoidosis (13). Besides distribution, further selectivity in action is reached by the process of flavonoid regeneration from the glucuronide metabolites. The liberation of the parent flavonoid molecule seems possible locally at the site where they should act, viz., a spot of inflammation where the beta-glucuronidase of neutrophils deals with the local de-conjugation of the glucuronide (14).

Flavonoids are well-known for their antioxidant action (15). It has even been reported that flavonoids can take over the role of physiological antioxidants like vitamin E (16). This general broad mode of action of flavonoids remarkably aligns with specificity, which is further illustrated by the exiting recent finding that flavonoids after being oxidized activate the transcription factor Nrf2. This is probably due to the thiol reactivity of the oxidized form of flavonoids (17). Toxicologists would in general classify thiol reactivity as a toxic process. However, in this case, it lengthens and intensifies the protective effect of flavonoids because Nrf2 activation gives induction of various protective cellular factors.

Flavonoids are regarded as widely available bioactives in the diet. They have been studied for decades and their multitude of activities is remarkable. Recent data indicate that their activity is at least partly due to their (toxic) reactivity. A clear example of the overlapping areas of toxicity and health.

## 8 Dietary components in combination with drugs

The focus in literature on food-drug interactions is the notion that these interactions result in negative effects in safety and efficacy of drug therapy, as well as in the nutritional status of the patient. Authors advise urging patients to inform their doctors and pharmacists about their food intake and dietary supplements so that these negative interactions can be avoided (18). Failure to identify and properly manage drug-nutrient interactions can lead to serious consequences (19). There is concern about interactions between herbal medicines or dietary supplements with conventional cytostatics in cancer patients (20).

In contrast to these negative aspects there are also examples of positive interactions between food or food components with drugs. Doxorubicin is a widely used anti-tumor drug. Its major side effect is a dose dependent cardiotoxicity. The flavonoid 7-mono-O-(β-hydroxyethyl)-rutoside (monoHER) can completely prevent this cardiotoxicity without interfering with the antitumor effect of doxorubicin in mice (21). The extrapolation of these data to humans is probably hampered because of different metabolism in mouse and human (22).

Besides this example in which a toxic side effect of drug is prevented with a food derived compound, it is also possible to stimulate the action of drugs. The efficacy of corticosteroids can be enhanced with flavonoids. This has broad clinical implications because desensitization to corticosteroids is a well-known phenomenon and might thus be prevented (23, 24). Moreover, a recent case report describes the facilitating effect of grapefruit juice in cortisol replacement therapy via modulation of drug metabolizing enzymes (25).

## 9 Vitamin B6 deficiency by high dose B6 supplementation

The water-soluble vitamin B6 functions as a coenzyme in many physiological reactions (26). Severe vitamin B6 deficiency is not very common. Alcoholics are at risk of deficiency because of low dietary intake and impaired metabolism of the vitamin. Neurologic symptoms might occur due to vitamin B6 deficiency. Paradoxically, supplementation with high doses of vitamin B6 may also lead to polyneuropathy (27). This rather enigmatic observation was recently explained (27). The major forms of vitamin B6 (also called B6 vitamers) are pyridoxine, pyridoxal, pyridoxamine, and their phosphorylated derivatives pyridoxine 5′-phosphate, pyridoxal 5′-phosphate, pyridoxamine 5-phosphate. Supplementation frequently occurs with pyridoxine, which has to be converted in the body into pyridoxal-phosphate, which is the active form. It was recently suggested that high levels of pyridoxine inhibit pyridoxal-phosphate dependent enzymes by competing with the active vitamer pyridoxal-phosphate. A high level of vitamin B6 in the form of pyridoxine thus inhibits the action of the active form of vitamin B6, i.e., pyridoxal-phosphate. A high dose of pyridoxine may give symptoms comparable to a vitamin B6 deficiency.

## 10 Botanicals: toxic or healthy?

Botanicals are substances derived from plants, algae, fungi or lichens. They are also called herbal dietary supplements. Although the consumer perception is that they are safer than conventional medicines, many adverse reactions by the products are described yearly. Worldwide discussion is ongoing which level of evidence is needed in order to provide market authorization for these products (28). In the USA these products are regulated by the “Dietary Supplement and Health Education Act” whereas in the European Union botanicals are sometimes brought to the food and sometimes to the pharmaceutical market. In the EU, pharmaceutical regulations have a provision that occasionally permits to include data on traditional use of botanicals in the approval process (28). In contrast, substantiation of health benefits of botanicals in the food market with evidence based on traditional use is complex. Authorization of a health claim in the EU is possible following the “Nutrition and Health Claim Regulation” (NHCR), which includes two randomized controlled trials Recognition to evidence based on traditional use, supporting a botanical health claim is still under debate (29, 30). Difficulties in using traditional data on heath promoting aspects is that modern botanicals should be used in a similar way with similar purpose as in traditional use. Extraction methods might be modernized yielding a more concentrated form of the botanical which may coincide with higher concentrations of potentially toxic contaminants. Moreover, simultaneous use with modern medicine may be different from the traditional situation lading to safety problems.

In general verification of safety steps like (i) characterization of the product; (ii) collection of bibliographic data; (iii) information from traditional use; (iv) data interpretation; (v) identification, interpretation and management control of risks, are important for botanicals (31).

## 11 The taste of healthy and toxic food

We started this reflection on heathy and toxic food, with tasters. Originally five basic tastes are discerned, viz., bitter, salty, sour, sweet and umami. It is generally thought that the bitter taste signals toxicity, alerting animals not to consume these bitter molecules. Unfortunately, detailed analysis showed that bitterness is not a very reliable marker for toxicity (32). In fact, extra-oral bitter taste receptors have other functions. Agonists for bitter taste receptors in lung tissue have for example been suggested to have therapeutic potential in the treatment of asthma (33).

Recently, a new model to describe mouthfeel has been presented (34). This model might be useful in situations where taste is distorted because of disease, age or drug use to optimize food perception. Although taste might not be the optimal tool to discern toxicity, it certainly is pivotal for good appetite and health.

## 12 Discussion

Some overarching concluding remarks on the notion of a sliding scale of healthy and toxic food based on these examples are possible. The awareness that a strict distinction between healthy and toxic food is not always possible will help the public to have less emotional fear for man-made chemicals. Simultaneously, the unsubstantiated dangerous believe in the safety of natural compounds will be challenged.

Hormesis, a biphasic response to toxic substances, needs more attention. This is not only important in risk assessment but also in explaining the health benefit of food ingredients like flavonoids.

Interaction of food with drugs should not be regarded from a negative viewpoint only. The positive aspects, i.e., protection against side effects of drugs or enhancing the efficacy of drugs needs receiving more attention.

The new definition of health, ability to adapt, leads to a paradigm shift in research on the influence of food on health. Recent developments in toxicology like the “adverse outcome pathways” encompassing novel molecular biology knowledge, may be helpful in understanding health and disease.

The “bitter taste” of toxins becomes sweeter if toxicity leads to healthiness.

## Author contributions

AB: Conceptualization, Writing – original draft, Writing – review & editing. KS: Writing – review & editing.
